# Unusual recurrent metastasizing benign breast papilloma: a case report

**DOI:** 10.1186/s13256-020-2354-7

**Published:** 2020-02-19

**Authors:** Amit L. Jain, Janice Mullins, Justin R. Smith, Poojitha Valasareddy, Emily Miller, Amina Chaudhry, Julie Ryder, Felicia Hare, Harsha Ranganath, Michael Berry, David Robins, Lee Schwartzberg, Gregory A. Vidal

**Affiliations:** 1 Internal Medicine Division, University of Tennessee Health Science Center, 956 Court Avenue, H314, Memphis, TN 38163 USA; 2 Division of Oncology, University of Tennessee Health Science Center, Memphis, TN USA; 3 West Cancer Center and Research Institute, Memphis, TN USA

**Keywords:** Breast papilloma, Breast malignancy, Benign papilloma

## Abstract

**Background:**

Papillary breast lesions may be benign, atypical, and malignant lesions. Pathological and clinical differentiation of breast papillomas can be a challenge. Unlike malignant lesions, benign breast papillomas are not classically associated with lymph node and distant metastasis. We report a unique case of a recurrent, benign breast papilloma presenting as an aggressive malignant tumor.

**Case presentation:**

Our patient was a 56-year-old postmenopausal African American woman who was followed in the breast clinic with a long history of multiple breast papillomas. She underwent multiple resections over the course of 7–9 years. After being lost to follow-up for 2 years, she once again presented with a slowly enlarging left breast mass. Subsequent imaging revealed a predominantly cystic mass in the left breast, as well as a suspicious hypermetabolic internal mammary node and a hypermetabolic nodule in the pretracheal space. Biopsy of the internal mammary node demonstrated papillary neoplasm with benign morphology and immunostains positive for estrogen receptor, progesterone receptor, and human epidermal growth factor receptor 2/Neu. Due to the clinical picture concerning for malignancy, the patient was then started on endocrine therapy with palbociclib and letrozole before surgery. She then underwent simple mastectomy and sentinel lymph node dissection with negative nodes and pathology once again revealing benign papillary neoplasm. She underwent adjuvant chest wall radiation for 6 weeks and received letrozole following completion of her radiation therapy. She was without evidence of disease 30 months after surgery.

**Conclusions:**

We present an unusual case of multiple recurrent peripheral papillomas with entirely benign histologic features exhibiting malignant behavior over a protracted period of many years, with an invasion of pectoralis musculature and possibly internal mammary and mediastinal nodes. Her treatment course included multiple surgeries (ultimately mastectomy), radiation therapy, and endocrine therapy.

## Introduction

Papillary lesions of the breast comprise a wide spectrum of tumors with varying clinical presentations and histopathological characteristics. That said, they constitute less than 2% of all breast lesions [1, 2]. The defining histology of these lesions is epithelial proliferation with fibrovascular core support with or without myoepithelial (ME) cells. These lesions may be benign, malignant, or atypical [3]. Papillomas with atypia are associated with up to a four times increased risk of malignant transformation, and unlike benign papillomas, malignant papillary lesions may be associated with lymph node and distant metastases [2, 4]. Benign breast papillomas presenting with local or distant metastases have been reported very rarely [5–8]. We present an unusual case of an African American woman with a recurrent, benign breast papilloma with malignant behavior.

## Case presentation

**Fig. 1:**
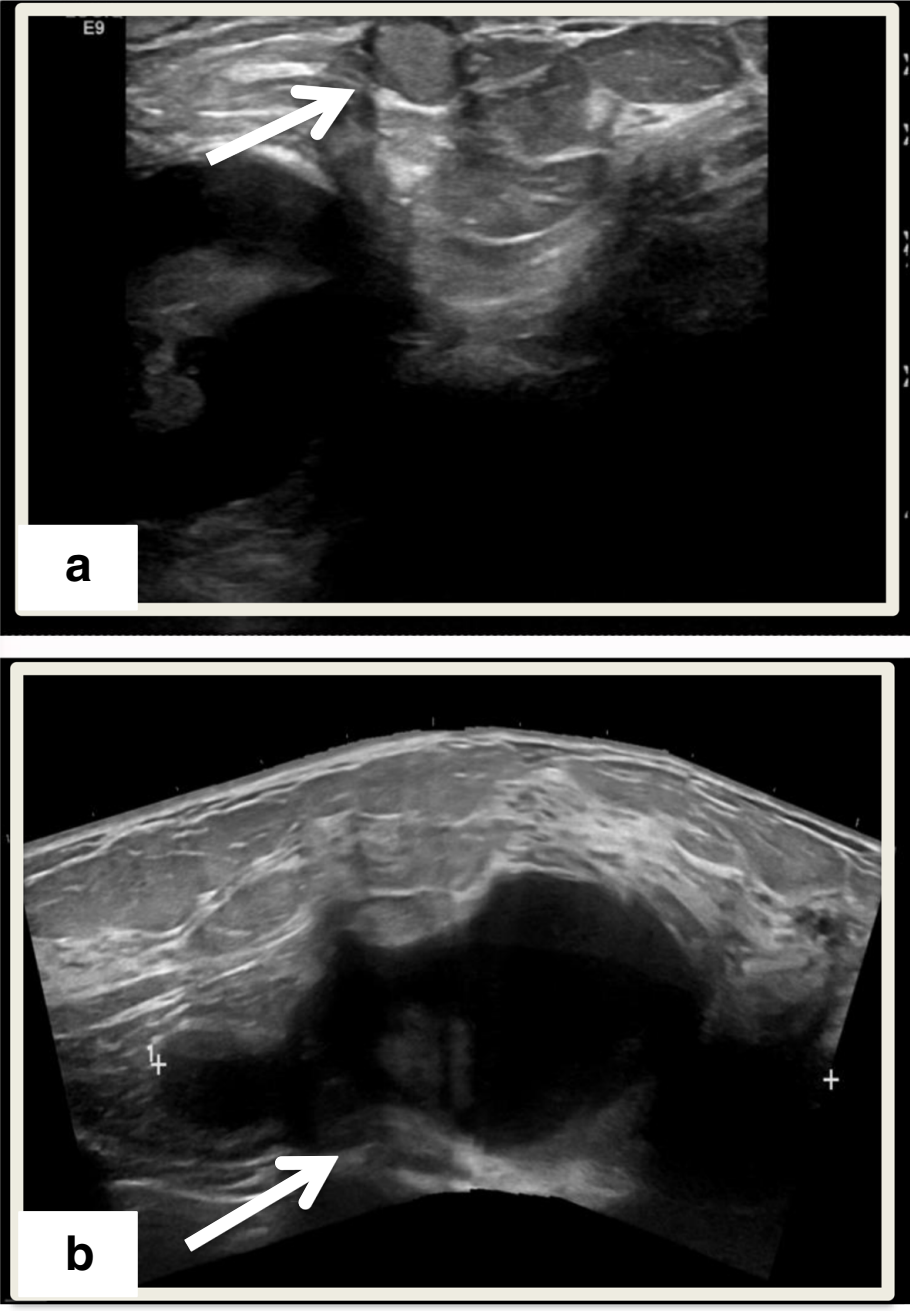
**a** Ultrasound image of 8-mm solid superficial mass located at six o’clock (white arrow). **b** Ultrasound image of 7-cm complex cystic mass located at one to three o’clock (white arrow)

**Fig. 2:**
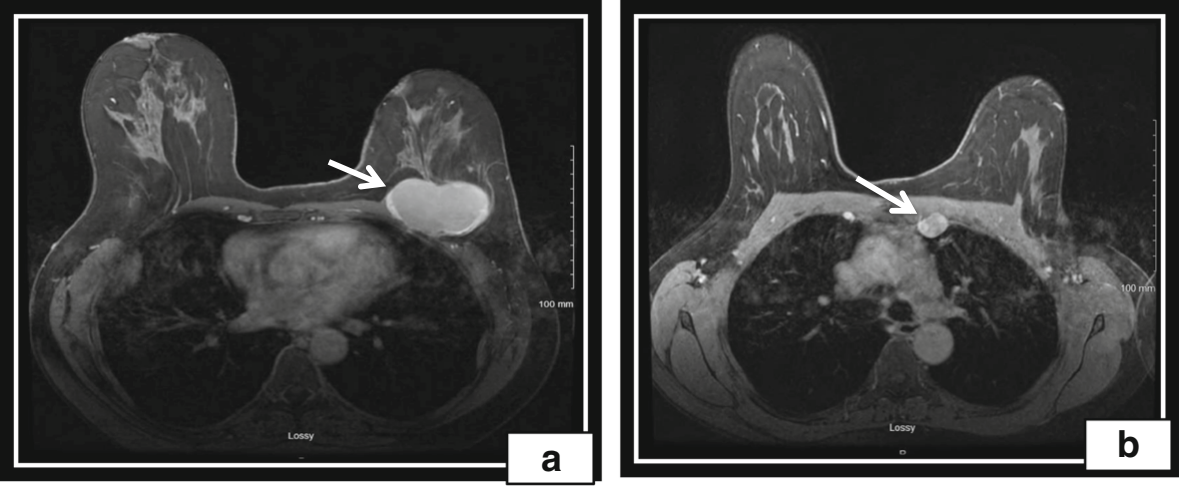
Magnetic resonance imaging of the breast with/without contrast enhancement. **a** Large cystic mass (white arrow) extending through the pectoralis muscle and containing a 1.8-cm area of neoplastic enhancement internally. This lesion was hypermetabolic on the positron emission tomographic scan. **b** An abnormal left internal mammary lymph node (white arrow) at the ​level of the sternomanubrial articulation. This was confirmed to be metastatic on the basis of biopsy

**Fig. 3:**
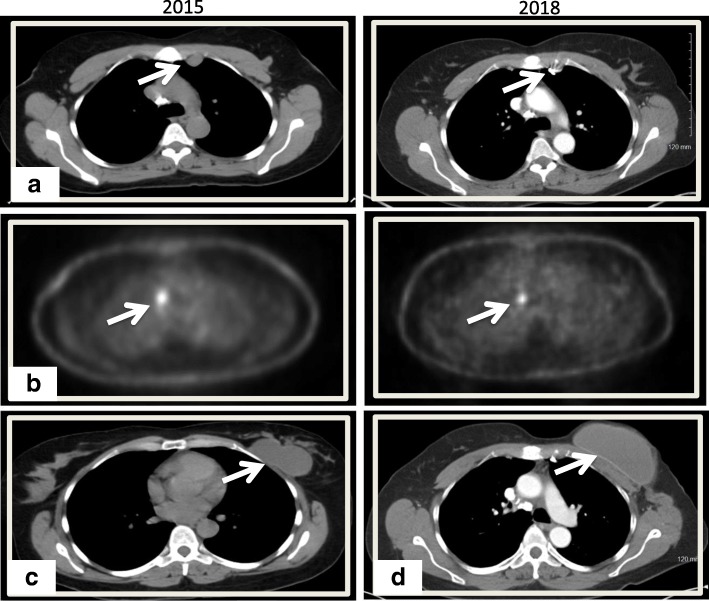
Comparison of positron emission tomographic/computed tomographic image from 2015 (prior to neoadjuvant antihormonal therapy and left breast mastectomy) versus follow-up image in 2018 (following left breast mastectomy). **a** Response was noted in the internal mammary lymph node following combination antihormonal therapy (*arrow*). **b**^18^F-fluorodeoxyglucose avid mediastinal lymphadenopathy with mild reduction in avidity following combination antihormonal therapy (*arrow*). Of note, no avidity was seen in the internal mammary lymph node, and minimal avidity surrounding the breast papillary mass was observed. **c** Mass in 2015 prior to antihormonal therapy and mastectomy. Note the size of this mass. **d** Reconstructed breast following mastectomy

Our patient was a 56-year-old postmenopausal African American woman with no past medical history who was previously treated at an outside oncology clinic for breast masses until 2010, when we first saw her. Her family history was negative for breast or ovarian carcinoma. She had a negative smoking history and endorsed drinking one drink per week. Per reports obtained, she first presented in 2006 with left breast lesions located in the upper inner breast that were documented as complicated cystic masses within the 9 o’clock and 9:30 positions on the basis of ultrasound (US). Subsequent US core biopsy in both areas revealed intraductal papilloma (IDP), and the patient was referred for a surgical consultation. No additional documentation of clinical visits was available until 1 year later. That documentation was in the form of a core biopsy pathologic report documenting the patient’s history of IDP at 9:00 and 9:30 positions as well as intraductal papillomatosis of the breast. A core biopsy taken at that time was from the left breast (location not mentioned) as well as the left axilla. The patient’s left breast showed fibrosis of mammary stroma including intralobular stromal sclerosis as well as microcalcifications in the lobular lumens. An axillary core biopsy confirmed IDP of the breast with stromal hyalinization as well as lymph node tissue adjacent to the papilloma. One month later, she underwent lumpectomy, with pathology reporting a 7-mm intracystic papilloma within a lymph node that was completely excised, as well as an epidermal inclusion cyst. The pathologist noted that the tumor was located near the periphery of a lymph node, probably arising in ectopic breast tissue in the capsular region. Approximately 11 months later, she developed another left breast mass. This was excised after a US-guided core biopsy once again revealed a benign IDP. The patient was then lost to follow-up at the outside clinic. She presented to our clinic 2 years later for an evaluation of a new left breast lesion. A bilateral diagnostic mammogram revealed two masses in the left breast, which were not well visualized, owing to heterogeneously dense breast tissue. Diagnostic US revealed a solid superficial mass measuring 0.81 × 0.76 × 0.81 cm corresponding to palpable findings also seen at the 6 o’clock position (Fig. 1a). Additionally, the patient had a large, complex cystic mass measuring 7 cm at the 1 to 3 o’clock position abutting the pectoralis muscle (Fig. 1a). A core biopsy of the 6 o’clock lesion was recommended. A US-guided, vacuum-assisted core biopsy of the 6 o’clock mass revealed an intracystic papillary neoplasm. Per the report, the patient denied nipple discharge, dimpling, thickening, redness of the skin, swelling, or tenderness at the time. A few weeks later, she underwent left breast lumpectomy with pathology revealing a complex cystic mass with fibrocystic changes at 1 to 3 o’clock and intraductal papilloma at 6 o’clock. The patient missed her 6-month follow-up mammogram. She returned 8 months later for a bilateral diagnostic mammogram, which showed a new 2.5-cm mass in the deep central aspect of her left breast at the 12 o’clock position. US showed a cystic mass measuring 3 cm and containing an intracystic solid component measuring 1 × 1 × 2 cm. No axillary or supraclavicular adenopathy was noted on the basis of imaging or physical examination. Her surgical team decided on left breast excisional biopsy with preoperative mammogram guidewire localization. Pathology revealed a benign papilloma measuring 1 cm, focally extending into skeletal muscle in the area adjacent to the previous biopsy site, but with negative margins and no signs of atypia. On the patient’s 6-month follow-up surveillance diagnostic mammogram, another new 3-cm density was noted at the 12 o’clock position. This was most consistent with a benign cyst and was aspirated. She was again lost to follow-up for more than 2 years until July 2015, when she presented with a 2-month history of a slowly enlarging left breast mass in the same region as her previous papillomas. A bilateral diagnostic mammogram with US showed a large mass at the 12 o’clock position measuring 7 × 2.5 cm. Her physical examination revealed that there were two areas of concern: first, a mass measuring 7.5 × 6.3 cm in the 1 o’clock position, and second, an area of nodularity measuring 4.6 × 3.1 cm in the 11 o’clock position. One month later, computed tomography (CT) of the chest and magnetic resonance imaging of the breast revealed a predominantly cystic mass with a solid component extending into the chest wall and approaching the pleural space (Fig. 2). These tests also revealed a suspicious internal mammary lymph node (Fig. 3a). A positron emission tomographic (PET)-CT scan showed a hypermetabolic nodule located in the pretracheal space (Fig. 3b) with a corresponding standardized uptake value (SUV) of 6.1 and multiple associated hypermetabolic internal mammary lymph nodes with the highest SUV of 6.0 and nodular hypermetabolic activity along the inferomedial aspect of the cystic mass (SUV, 2.7).

Her case was discussed at the multidisciplinary breast tumor board, and the recommendation was to proceed with a biopsy of the left internal mammary lymph nodes. Core biopsy revealed a papillary neoplasm with benign morphology with immunostains positive for estrogen receptor (ER) at 99%, positive for progesterone receptor (PR) at 85%, HER2/neu 1+, and a Ki67 proliferation index of 6%. An independent external pathologist agreed with the finding of histologically benign papilloma. The patient sustained a biopsy-related internal mammary artery injury and as a result developed a hemothorax requiring video-assisted thoracoscopic surgery.

**Fig. 4:**
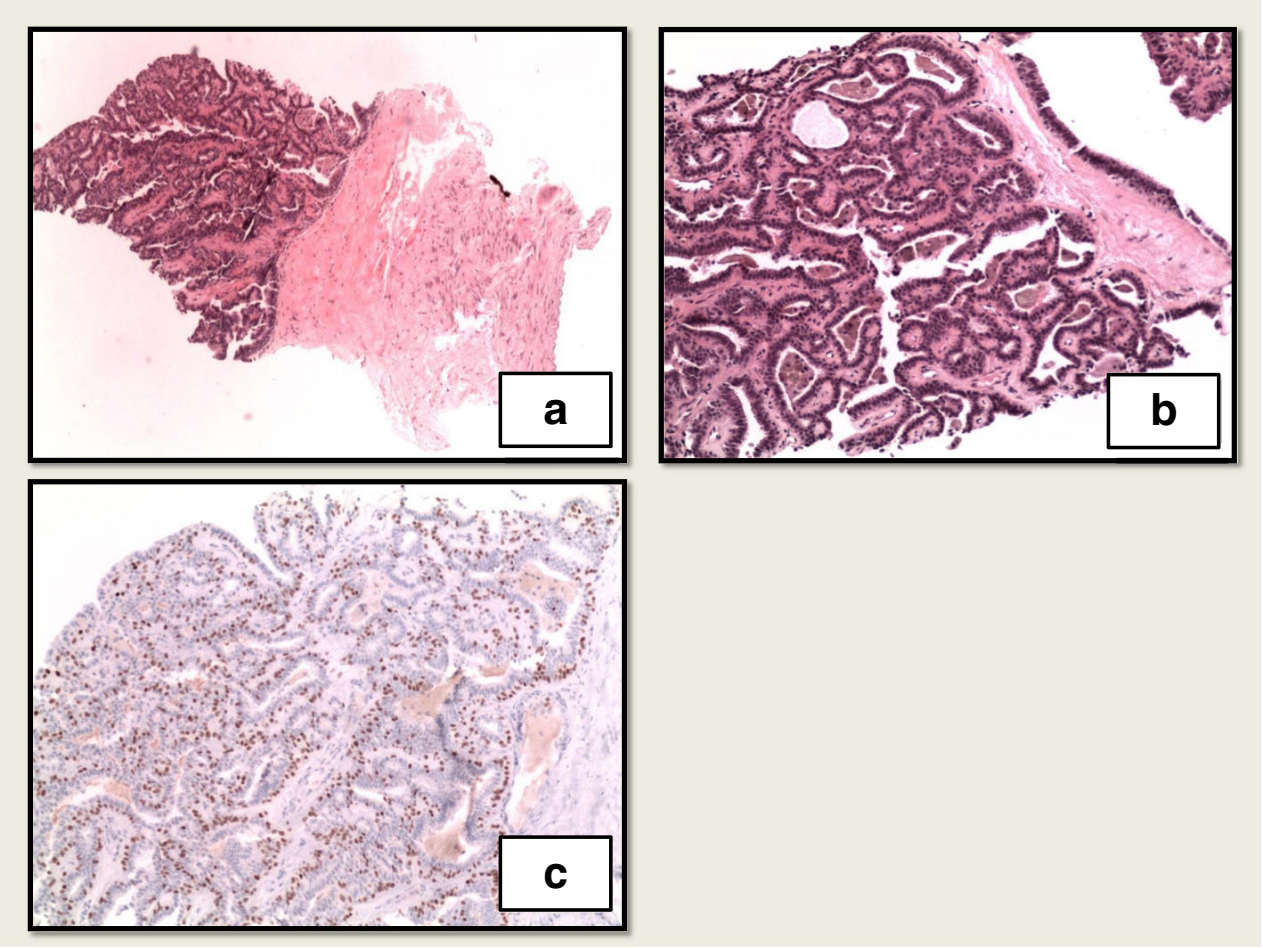
Left internal mammary node core biopsy. **a** Well-demarcated papillary neoplasm in fibroadipose tissue. No lymph node structure was identified. **b** Medium-power view of papillary neoplasm. Prominent fibrovascular cores with bland columnar cell lining. **c** p63 immunohistochemical stain highlights the intact myoepithelial layer beneath the columnar lining

**Fig. 5:**
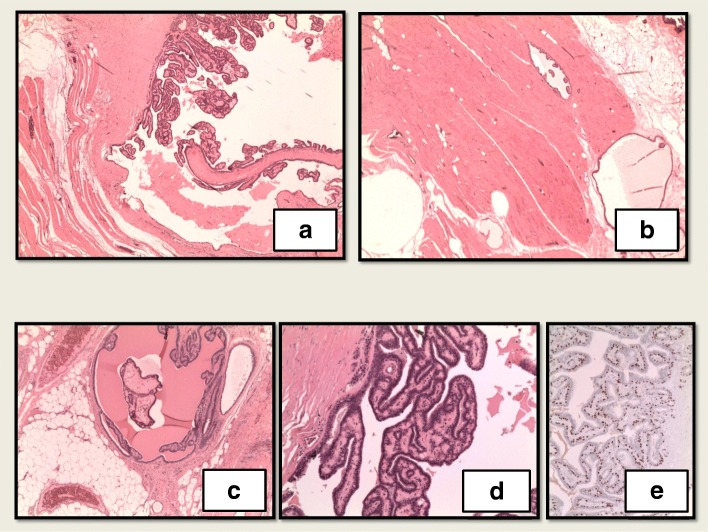
Mastectomy. **a** Cystic papilloma within deep pectoralis muscle. **b** Papilloma in pectoralis muscle. **c** Papilloma in deep adipose tissue. **d** Papilloma with bland features and fibrovascular cores. **e** p63 immunostain highlights intact myoepithelial layer throughout

Upon recovery from the hemothorax, the patient was referred to the medical oncology department of our hospital. Given the malignant behavior of her tumor, a recommendation of aggressive local control was made. She was started on endocrine therapy with palbociclib and letrozole as a neoadjuvant strategy. Repeat PET-CT following 4 months of combination antiestrogen therapy demonstrated near-complete resolution of metastatic internal mammary lymph nodes (white arrows in Fig. 3a) and reduced size and avidity of the paratracheal nodes (arrows in Fig. 3b). A physical examination did not show any significant changes in the size of the left breast mass. She went on to complete 6 months of neoadjuvant therapy. The primary lesion demonstrated minimal clinical response after 6 months of combination endocrine therapy, and then she underwent a left simple mastectomy and sentinel lymph node biopsy. Once again, the pathology revealed a 7.1-cm papillary neoplasm described as microscopically bland and mitotically inactive, with a retained ME layer. Several similar-appearing satellite papillomatous lesions were also seen within the skeletal muscle and deep adipose tissue. Margins and all five sentinel lymph nodes were negative (Figs. 4 and 5).

Given the extent of her local involvement and history of recurrent disease, she underwent adjuvant chest wall radiation for 6 weeks, followed by adjuvant endocrine therapy with letrozole. Six months after mastectomy, a repeat PET-CT scan showed no evidence of disease. She continues to undergo surveillance CT of the chest and mammography of her right breast. One year after her mastectomy, she underwent left breast reconstruction (Fig. 3d). She remained without evidence of disease 2.5 years after mastectomy and continued on endocrine therapy during that time.

## Discussion

Papillary lesions, per the World Health Organization classification, are classified as benign papillomas, papillomas with atypical ductal hyperplasia (ADH), papillomas with ductal carcinoma *in situ* (DCIS), intraductal papillary carcinoma (IPC), encapsulated papillary carcinoma (EPC), solid papillary carcinoma (SPC), and invasive papillary carcinoma [9]. Clinically, SPC and EPC are regarded as variants of IPC [3]. On occasion, morphological distinction between a benign and a malignant papillary lesion can be challenging. Although the absence of an intact ME cell layer in the fibrovascular core suggests carcinoma, its presence does not always exclude malignancy [10–13]. Invasive growth and high-grade nuclei support carcinoma [14]. Also, very few cases have been reported of metastases from benign papillomas, and all of them were reported as metastases to the axillary lymph node [5–8]. To our knowledge, we report the first case of a benign breast papilloma extending into the chest wall and also involving the internal mammary node.

Papillary lesions are categorized as central (involving large, lactiferous ducts) or peripheral (involving terminal ductal lobular unit), based on location [15]. Central IDPs are classically solitary and are more common than peripheral IDPs, which usually are multiple. Solitary and central IDPs are associated with atypia or DCIS less frequently than multiple and peripheral IDPs are [16]. Further, the presence of ADH or DCIS in papillomas is associated with a risk of subsequent malignancy, which varies widely (7–67%) [1, 17–20]. Our patient’s central intracystic papilloma was not associated with atypia or DCIS, but the clinical behavior was suspicious for a malignant papillary lesion. Of note, IPCs are rare, but they are an essential part of the differential diagnosis of papillary lesions [21].

Interestingly, papillary lesions are innately friable and susceptible to epithelial displacement following a fine-needle aspiration or core-needle biopsy. The epithelial displacement can occur into the biopsy site, lymphatic channels, or axillary lymph nodes. Understanding this phenomenon helps to prevent misdiagnosis and differentiate benign from potential metastatic lesions [22, 23].

Central IDPs can occur at any age, but they are prevalent between the ages of 40 and 60 years [17]. Similar to our patient, patients with central IDPs classically present with a palpable breast mass with or without nipple discharge and a well-defined mass on a mammogram. In contrast, peripheral IDPs occur in younger patients and are often clinically occult and diagnosed incidentally as microcalcifications during screening mammography [24].

IPC generally occurs in older patients. About half of these cases arise centrally, commonly have associated bloody nipple discharge, and 90% have a palpable mass [25]. On mammography, IPCs appear as rounded, well-circumscribed lesions. Patients with IPC are rarely associated with lymph node involvement and distant metastasis and generally have an excellent prognosis, with a 5-year survival greater than 80% [3, 26, 27].

The treatment strategy for papillary lesions of the breast is debatable because there is always a high suspicion of harboring malignant lesions. For example, atypical papillomas are associated with high rates of malignant upgrades (as high as 42%), and hence surgical excision is the current standard of care [28–30]. By contrast, benign papillomas demonstrate malignant upgrade at rates less than 10%, and management can vary from conservative radiologic follow-up to surgical excision [31, 32]. However, many experts also advocate complete surgical excision due to sampling errors at the time of the biopsy and tumor heterogeneity with atypical or malignant foci [19, 33–35]. Local recurrence of solitary papilloma after surgical excision is also uncommon, occurring in less than 10% of cases [27, 36]. Compared with our patient’s case, multiple recurrences are rare, even with a malignant papillary lesion. As a result, surveillance imaging and follow-up per institutional and national guidelines are recommended.

Given its low malignant potential and low proliferative index, chemotherapy is not recommended for the treatment of IPC [37]. The majority of IPC cases express ER and PR. Adjuvant endocrine therapy, therefore, remains an option. For benign papillary lesions, secondary systemic therapies are not recommended, although primary prevention with antiestrogen therapy may be recommended on the basis of a patient’s risk assessment [38]. In our patient, the pattern of recurrence, including muscle invasion, tumor size, and intramammary nodal involvement, was atypical, pointed to a more malignant tumor behavior, and was therefore approached in a more aggressive manner. No evidence exists for the use of combination antiestrogen therapy or CDK4/6 inhibitor therapies for the treatment of potentially curable invasion or benign papillary carcinomas. Ultimately, this patient appears to have benefited from this approach.

## Conclusion

We present an unusual case of a patient with multiple recurrent peripheral papillomas with entirely benign histologic features exhibiting malignant behavior over a protracted period of many years, including invasion of pectoralis musculature, internal mammary, and possibly paratracheal lymph nodes. Her treatment course included multiple surgeries (ultimately mastectomy), radiation, and endocrine therapy. The patient was disease-free 32 months after mastectomy.

## Data Availability

Not applicable.
